# Flow of heterotrophic production in oligotrophic ocean waters

**DOI:** 10.3389/fmicb.2025.1530627

**Published:** 2025-03-12

**Authors:** Afrah Alothman, Carlos M. Duarte, Mohammed Ali Qurban, Susana Agustí

**Affiliations:** ^1^Marine Science Program, Biological and Environmental Science and Engineering Division, King Abdullah University of Science and Technology (KAUST), Thuwal, Saudi Arabia; ^2^National Center for Wildlife, Riyadh, Saudi Arabia

**Keywords:** bacterial production, glucose uptake, primary production, carbon transfer, oligotrophic waters, stable isotopes, microbial loop

## Abstract

In oligotrophic ecosystems, bacterial production (BP) via the microbial loop and grazing processes plays a crucial role in carbon transfer (CT) to higher trophic levels. However, there studies quantifying CT from bacteria to the marine food web are limited. In this study, we used ^13^C-isotope tracers and cavity ring-down spectroscopy to measure primary production (PP), BP, bacterial respiration (BR), and CT within the microbial food web in oligotrophic waters. Our results revealed that the BP rate, ranging from 0.02 to 4.93 μg C L^−1^ d^−1^, was significantly lower than the total PP, which ranged from 2.69 to 16.71 μg C L^−1^ d^−1^. Our findings indicate that grazing of bacteria in the Red Sea is substantial. The removal of grazers through prefiltration lead to a 9.5-fold increase in BP rates, rising from 0.37 ± 0.04 μg C L^−1^ d^−1^ to 3.52 ± 1.04 μg C L^−1^ d^−1^ at the stations analyzed. This significant increase suggests that a large portion of bacterial carbon is rapidly transfer to higher trophic levels via grazing. In addition, carbon transfer (CT) to the food web, measured in size fractions above picoplankton (>1.2 or > 3 μm), accounted for an average of 72.7 ± 4.0% of the net bacterial production (Net BP = BP + CT), underscore the crucial role of grazers in bacterial carbon cycling. This transfer increased significantly with increasing temperatures, highlighting the enhanced role of the microbial loop in CT during warmer conditions. We found that at some stations, a large proportion of the carbon assimilated by bacteria was used for respiration, averaging 1.37 ± 0.54 μg C L^−1^ d^−1^. This high respiratory demand of bacterial cells in oligotrophic waters may explain the low bacterial growth efficiency (BGE) of 9.7% ± 1.0% observed in our study, along with the significant correlation between BP and BGE. Our findings demonstrated that BP effectively transfers carbon through the microbial loop to higher trophic levels in the oligotrophic and warm waters of the Red Sea.

## 1 Introduction

Heterotrophic bacteria play a crucial role in the transfer and cycling of organic matter and nutrients within ecosystems, significantly influencing the structure and function of the marine food web (Azam et al., 1983; Ducklow and Kirchman, 2000). On average, half of oceanic primary production (PP) is channeled as dissolved organic matter to bacteria (Cole et al., 1988), with this fraction being even greater in the oligotrophic oceans. In these regions, processes such as exudation, viral lysis (Lara et al., 2017), and phytoplankton cell lysis (Agustí et al., 1998; Agusti and Duarte, 2013) release substantial amounts of carbon as dissolved organic carbon (Thornton, 2014). Heterotrophic bacteria take up organic compounds to support their growth and metabolism, serving two roles: decomposing organic matter into CO₂ and inorganic nutrients, and acting as components of the microbial food web by channeling organic carbon to protists and metazoans. These bacteria are key contributors to plankton community respiration (del Giorgio and Duarte, 2002), accounting for an estimated 70–92% of particulate organic carbon (POC) remineralization in the North Atlantic Ocean alone (Giering et al., 2014), which supports recycled PP. Additionally, heterotrophic bacteria convert dissolved organic carbon (DOC) into POC, facilitating the transfer of bacteria to heterotrophic protists (Calbet and Landry, 2004) and higher trophic levels (Mostajir et al., 2015).

Assessments of bacterial growth efficiency (BGE) (Del Giorgio and Cole, 1998) showed that most heterotrophic BP is remineralized, with only a small percentage contributing to biomass entering the food web. This trend is particularly evident in the warm, oligotrophic ocean, where BGE is reduced (Berglund et al., 2007; López-Urrutia and Morán, 2007; Vazquez-Dominguez et al., 2007; Sarmento et al., 2010; Lara et al., 2013). Indeed, in oligotrophic waters, heterotrophic bacterial respiration (BR) can approach or even exceed PP rates (Del Giorgio et al., 1997), while the biomass of heterotrophic bacteria has been observed to match or exceed that of phytoplankton (Fuhrman and Ferguson, 1986; Cho and Azam, 1990; Gasol et al., 1997). Consequently, the oligotrophic ocean is often considered heterotrophic (Duarte et al., 2013) or in a close metabolic balance (Williams et al., 2013). However, in a seminal paper, Azam et al. (1983) introduced the microbial carbon loop concept, suggesting that grazing on heterotrophic bacteria by heterotrophic flagellates and microzooplankton prevents organic carbon loss by looping bacterial carbon into the conventional planktonic food chain. The observation that oligotrophic oceans support a substantial biomass of mesopelagic fish (Irigoien et al., 2014) challenges the notion that their food web is inefficient in channeling carbon upward. This suggests that the microbial loop may transfer carbon up the oligotrophic ocean food web more than previously thought. However, data on carbon transfer (CT) to the food web through DOC utilization by heterotrophic bacteria are limited and mostly derived from indirect measures of heterotrophic BP and protist grazing rates (Fuhrman and Azam, 1982; Kirchman et al., 1982; Kirchman et al., 1985).

Nonetheless, some studies have employed labeled carbon, such as ^13^C-glucose (Koshikawa et al., 1996; Koshikawa et al., 1999) or ^14^C-glucose uptake (Wylie and Currie, 1991), to assess DOC transfer to grazers through heterotrophic bacteria. In this study, we examined the role of BP in transferring carbon through the microbial loop to higher trophic levels in the oligotrophic Red Sea (Supplementary Figure S1). The Red Sea is a tropical and oligotrophic ocean basin with relatively warm and isothermal subsurface waters ranging from 21°C to 33°C (Morcos, 1970; Halim, 1984; Shaltout, 2019). Its phytoplankton community is dominated by pico- and nano-phytoplankton (Stambler, 2006; Pearman et al., 2016; Kheireddine et al., 2017). We used the ^13^C stable isotope tracers’ technique and cavity ring-down spectroscopy (CRDS) to measure the PP and BP rates in the surface waters of the eastern Red Sea (Supplementary Figure S1). Additionally, we employed fractionated filtration to assess CT to the food web (Supplementary Figure S1). Our results revealed that CT significantly exceeded BP, accounting for approximately 72.73% of net BP (BP + CT). This supports the hypothesis that BP is efficiently transferred to higher trophic levels in oligotrophic systems. Furthermore, the results showed that CT increased with rising temperature, highlighting the enhanced role of CT via the microbial loop in warm waters.

## 2 Methods

### 2.1 Field sampling and processing

**Figure 1:**
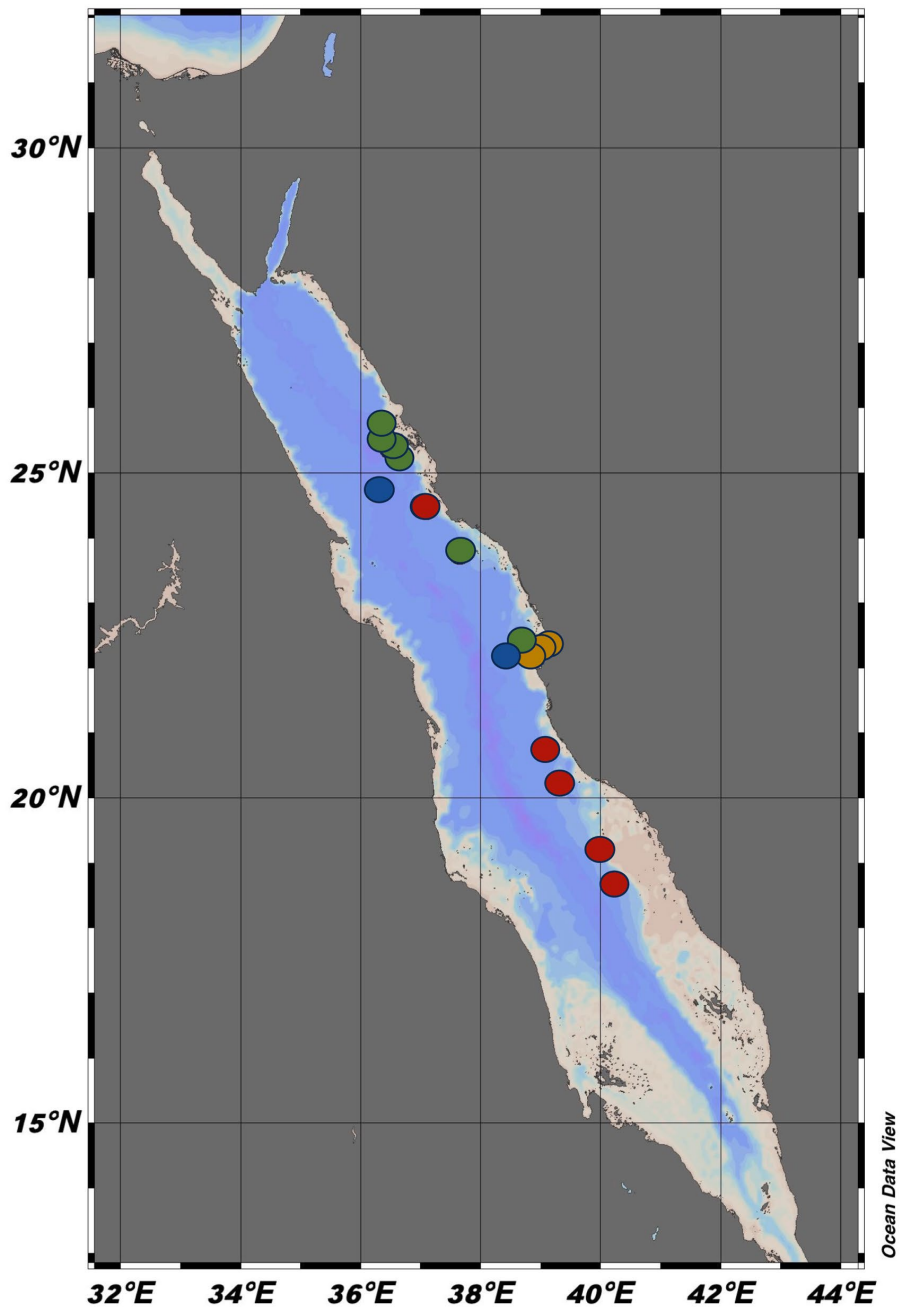
The 16 sampling stations along the Eastern Red Sea, where the study was conducted between 2019 and 2022. The red dots showed the Deep Cruise (DC), the green dots represent the Deep Coral Survey cruise (DCS), the blue dots represent the Red Sea Decade Expedition (RSDE), and the orange dots represent the coastal stations. Each dot represents the stations where bacterial production and carbon transfer to the microbial food web were measured. The blue dots specifically represent the stations where bacterial production was further measured after grazer removal (BP without grazers) in addition to bacterial production with grazers and carbon transfer. The green dots represent the stations where bacterial respiration (BR) and bacterial growth efficiency (BGE) were also measured. The map was generated using Ocean Data View software (Version 5.6.5).

We studied 16 sampling stations during three oceanographic expeditions along the Red Sea—Deep Cruise (DC, April 3–8, 2019), Deep Coral Survey (DCS, January 15–23, 2020), Red Sea Decade Expedition (RSDE, 2022), onboard R/V *Thuwal* (DC and DCS), and R/V *OceanXplorer* (RSDE) (Figure 1). Additionally, water samples were obtained from three central Red Sea coastal stations, including pelagic waters, the reef Abu Shusha, and a coastal lagoon near the King Abdullah University of Science and Technology (KAUST) (Figure 1). During the cruises, we gathered the environmental vertical profiles of each station, including *in vivo* fluorescence (WetLabs ECO FL fluorometer), temperature, and salinity. These measurements were obtained using a Sea-Bird SBE 911plus CTD profiler (Sea-Bird Electronics, Bellevue, WA, USA) attached to a rosette sampling system. At the coastal stations, temperature, and salinity were recorded for 15 min using an Idronaut Ocean Seven 305plus CTD. Surface water samples were collected from each station using 10-L Niskin bottles connected to a rosette system (DC and DCS) or deployed manually (RSDE and coastal stations).

### 2.2 Chlorophyll-*a* and nutrient concentrations

To measure chlorophyll-*a* concentration (Chl-*a*), water samples were collected from a depth of 5 m at each station and filtered using 25 mm Whatman GF/F filters with a pore size of 0.7 μm. The resulting filtrates were extracted in 90% acetone and stored in the dark for 24 h, following the methodology outlined by Prabowo and Agusti (2019). The extracted pigment was assessed using a Trilogy Fluorometer with a CHL-NA module (Turner Designs, San Jose, CA, USA) calibrated against pure Chl-*a* (Prabowo and Agusti, 2019). Additionally, 50 mL water samples were frozen for later analysis of inorganic nutrient concentrations using standard autoanalyzer methods outlined by Hansen and Koroleff (1999), with a Segmented Flow Analyzer (SEAL AA3 Analytical Inc., WI, USA).

### 2.3 Picophytoplankton and heterotrophic bacterial abundances

We quantified picophytoplankton and heterotrophic bacterial cells in the surface seawater for each sample. From each sample, we collected 1.8 mL into small cryovials, fixed them with 25% glutaraldehyde, rapidly froze them using liquid nitrogen, and stored them at −80°C for later analysis. To determine the bacterial abundance, we stained the heterotrophic bacterial samples with a 1:100 SYBR Green I intermediate solution. We analyzed the abundance of picophytoplankton and bacteria using flow cytometry, employing either a FACSCanto II (Becton Dickinson) or a Flow Cytometer Cube 8 (Sysmex), following the methodology described by Gasol and Morán (2015).

### 2.4 Dissolved inorganic carbon

We analyzed Delta (δ^13^C) signals of dissolved inorganic carbon (DIC) in both natural water samples and those enriched with ^13^C-sodium bicarbonate (NaH^13^CO_3_). Samples were collected in 15-mL glass vials and treated with 0.05% mercuric chloride (Cl_2_Hg, Sigma-Aldrich) to preserve and inhibit biological activity. Subsequently, the samples were stored in a dark and cold environment until laboratory analysis. DIC quantification was performed using an AutoMate Prep Device connected to Picarro’s LIAISON interface and the IsoCO2 WS-CRDS system (Santa Clara, California, USA).

### 2.5 Primary production

PP was measured using a stable isotopic tracer (^13^C-NaHCO_3_, Sigma-Aldrich) at a final carbon concentration of approximately 153 μmole ^13^C L^−1^ following the PP^13^C method described by López-Sandoval et al. (2019). Before isotopic enrichment, the water samples were prefiltered through a 100 μm mesh to remove larger zooplankton and copepods. The samples for measuring PP were then distributed into three replicated acid-washed 2-L (DC, DCS, and coastal) or 500 mL (RSDE) transparent polycarbonate bottles (PC), along with one dark PC bottle for dark uptake correction. The samples were enriched with NaH^13^CO_3_ and placed in tanks on the deck of the vessels, where circulating surface seawater maintained *in situ* sea-surface temperatures and exposure to natural solar radiation. The PP bottles were covered with neutral-density nets to reduce light intensity, simulating the light conditions at a depth of 5 m. Coastal water samples were incubated in outdoor tanks at the Coastal & Marine Resources Core Lab at the KAUST. After 4–6 h of incubation, the content of each bottle was sequentially filtered through 25-mm diameter filters with varying pore sizes to obtain the PP in the pico-sized (1.2–3 μm) fraction and larger plankton (nano- and micro-phytoplankton). We used precombusted 3 μm silver membranes (STERLITECH) to capture the larger plankton fraction of the community for the DCS and RSDE samples, while we used precombusted Whatman GF/C filters (nominal pore size of 1.2 μm) for the DC and coastal station samples. Each filtered water sample was passed through precombusted 0.2 μm silver membranes (STERLITECH) to quantify the PP in the picophytoplankton fraction of the community. The silver filters were chosen due to their availability in different pore sizes (0.2 μm and 3 μm), which are suitable for fractionation, as well as their low background carbon content, ensuring high-temperature compatibility for stable isotope analysis.

A surface seawater sample was collected at each station to measure the carbon content and the natural isotopic composition following a similar fractionation method described previously. After filtration, all filters were placed in small glass Petri dishes and treated with 150 μL of 50% HCL to eliminate inorganic carbon, dried overnight, and stored at −20°C until analysis.

The carbon content and the *δ* isotopic values of the filters were evaluated using a combustion module coupled with a cavity ring-down spectroscopy analyzer (CM-CRDS-G2201-I, Picarro). Each filter underwent combustion for 720 s, converting the sample into the required gas (CO_2_) through rapid combustion, after which the gas was transferred to the isotopic analyzer to measure the ^13^C/^12^C ratio (δ^13^C). Spectrum peaks were generated within the cavity based on the measured wavelengths absorbed by the target gases (^13^CO_2_ and ^12^CO_2_), with each peak corresponding to the ^13^C and ^12^C concentrations (López-Sandoval et al., 2019).

Before analyzing the samples, the CRDS Picarro instrument was calibrated using Vienna Pee Dee Belemnite (VPDB) standards from the International Atomic Energy Agency (IAEA), including IAEA-CH-6, C3, and 303B, with *δ*^13^C values of −10.45‰, −24.72‰, and + 450‰, respectively. Standards from the Reston Stable Isotopic Laboratory (United States Geological Survey, USGS) (López-Sandoval et al., 2019) were also used: USG62 (−14.79‰), USG40 (−26.39‰), and USG41a (+36.55‰).

After the incubation period (4–6 h), the ^13^C and ^12^C isotopic masses from the ^13^C-enriched samples were measured to calculate PP^13^C (μg C^−1^ h^−1^) for both size fractions of the community (PP^13^C, > 3 μm and PP^13^C, < 3 μm) using the ^13^C-CRDS-PP approach (López-Sandoval et al., 2019). Specifically, we calculated the PP^13^C, > 3 μm and PP^13^C, < 3 μm as the isotopic shift of POC from the light-incubated samples (*δ*^13^C_POC-Light_) relative to the dark isotopic composition of the samples (δ^13^C_POC-Dark_). Moreover, we also calculated the isotopic shift of the enriched DIC (δ^13^C_DIC-Enriched_) relative to the natural DIC samples (δ^13^C_DIC-Natural_). Subsequently, we converted the production to carbon uptake rates by considering the POC measured at the end of incubation in light-enriched samples per filtered volume (v) in liters (POC-μg C L^−1^). The carbon fixation rate was then calculated per unit time (t, hours of incubation) using Equation 1 below:


(1) $$PP^{13}C=\left[\frac{\left(\delta^{13}C_{POC-\mathrm{Light}}-\delta^{13}C_{POC-\mathrm{Dark}}\right)}{\left(\delta^{13}C_{DIC-\mathrm{Enriched}}-\delta^{13}C_{DIC-\mathrm{Natural}}\right)}\times POC\right]/V/t$$

We converted the PP data into daily carbon uptake rates (PP^13^C μg C L^−1^ d^−1^), assuming a local photoperiod of 12 h of light and considering our incubation time of 4 h. Total PP (TPP) was then calculated by summing the PP from the fraction larger than 3 μm (PP^13^C, > 3 μm) captured on the 3 μm filter, and the PP from the fraction smaller than 3 μm (PP^13^C, < 3 μm) captured on the 0.2 μm filter after passing through the 3 μm filter, using Equation 2 below:


(2) $$TPP=\left(PP^{13}C{,}_{>3\;\mathrm{\mu m}}+PP^{13}C{,}_{<3\;\mathrm{\mu m}}\right)$$

### 2.6 Bacterial production and carbon transfer to the food web

We quantified BP using a stable isotopic tracer (D-Glucose-^13^C_6_, Sigma-Aldrich) at final carbon concentrations of 100 nM for DC and coastal stations (Koshikawa et al., 1999) and 75 nM for DCS and RSDE. This process was coupled with the CRDS Picarro instrument, following the methodology outlined by Alothman et al. (2023). We measured BP as ^13^C-glucose uptake rate (GUR^13^C), expressed in μg C L^−1^ h^−1^, as detailed by Middelburg et al. (2000).

All surface water samples were prefiltered using a 100 μm mesh to remove larger zooplankton and copepods before the isotopic addition. For the ^13^C-glucose uptake rate measurements, the water samples were distributed in three replicated dark acid-washed 2-L (DC, DCS, and coastal) or 500 mL (RSDE) PC bottles. Two water samples from the selected stations (RSDE, Figure 1) were further prefiltered through 3 μm pore size filters before incubation to remove small grazers, such as flagellates and ciliates. This prefiltration enabled us to examine how removing various-sized grazers (zooplankton, copepods, dinoflagellates, and ciliates) affected BP. Following the ^13^C-glucose enrichment, all dark PC bottles were incubated for 4–6 h in tanks connected to a circulating seawater system to maintain *in situ* temperature while being placed on the deck of the vessels.

At the end of the incubation, the contents of each bottle were serially filtered through 25-mm diameter filters with pore sizes similar to those described for PP (Section 2.5). The plankton fraction size of the community was collected on precombusted 3 μm silver membranes for the DCS and RSDE samples and on precombusted Whatman GF/C filters (1.2 μm pore size) for the DC and coastal station samples. The filtrate water was then filtered through precombusted 0.2 μm silver membranes to quantify the glucose uptake in the bacterial fraction. For the grazing experiments at the two stations, each water content at the end of the incubation was collected on the precombusted 0.2 μm silver membranes. At each station, one water sample was used to measure carbon content and natural isotopic composition, following the same fractionation methods described previously. After filtration, the filters were placed in glass Petri dishes with 150 μL of 50% HCL to remove inorganic carbon. All filters were dried overnight and stored at −20°C until laboratory analysis.

BP was measured as the bacterial fraction size of the community collected on 0.2 μm filters from all incubated water samples. We further captured the larger fraction size on the 3 μm silver filters to measure the CT to the higher trophic levels of the microbial loop. Both BP and CT—expressed as ^13^C-glucose uptake rate (μg C L^−1^ h^−1^) were determined by calculating the difference between the ^13^C fraction in the natural isotopic composition sample (F_Natural_) and the ^13^C fraction in the enriched sample (F_Enriched_), as shown in Equation 3 below:


(3) $$GUR^{13}C=\left[\left(F_{\mathrm{Enriched}}-F_{\mathrm{Natural}}\right)\times POC\right]/V/t$$

Here, *F* = ^13^C/(^13^C + ^12^C), which can also be expressed as R / (R + 1). R is the carbon isotope ratio derived from the measured *δ* ^13^C values, calculated using Equation 4 below (derived from Middelburg et al., 2000):


(4) $$R=\left(\frac{\delta^{13}C}{1000}+1\right)\times \mathrm{VPDB}\;\left(\mathrm{Vienna}\;\mathrm{Pee}\;Dee\;\mathrm{Belemnite}\right)$$

Here, VPDB = 0.0112372. The uptake rate was also determined by accounting for the POC measured in the samples at the end of the incubation, per time unit (t, hours of incubation), and per volume (v) filtered in liters (L). Both BP and CT rates were reported per day, assuming a 24-h cycle for bacterial carbon production and transfer.

### 2.7 Bacterial respiration and bacterial growth efficiency

At the end of the ^13^C-glucose dark incubation period at six sampling stations (DCS stations, Figure 1), we collected 15 mL of water samples to quantify the change in DIC (δ ^13^C-glucose_DIC-Enriched_) and assess BR. These measurements were conducted exclusively on the <3 μm fraction, ensuring that BR estimates reflect bacterial activity rather than community respiration. These samples were processed similarly to the natural (δ ^13^C-glucose_DIC-Natural_) and enriched DIC samples described in Section 2.4. We calculated BR using Equation 5 below:


(5) $$BR=\left(\delta^{13}\mathrm{Cglucos}e_{DIC-\mathrm{Enriched}}-\delta^{13}C_{DIC-\mathrm{Natural}}\right)\times POC/t$$

Here, POC is the particulate organic carbon obtained in the Picarro measurement at the end of the dark incubations with glucose. The BR rate was then expressed per unit of time (t, hours of incubation).

We also measured the BGE to relate BP and BR. BGE is the amount of new bacterial biomass synthesized per unit of assimilated substrate (Del Giorgio and Cole, 1998). It was calculated as the total BP biomass, represented by BP and CT (net BP), relative to the sum of net BP and BR (gross BP), as shown in Equation 6 below:


(6) $$BGE=\frac{\left(BP+CT\right)}{\left(BP+CT+BR\right)}$$

or


$$BGE=\frac{\left(Net\;BP\right)}{\left(\mathrm{Gross}\;BP\right)}$$

### 2.8 Statistical analysis

Statistical analysis was conducted using JMP PRO 16 software (JMP^®^, Version 16.0, SAS Institute Inc., Cary, NC, 1989–2019). Statistical significance was set at *p* ≤ 0.05. We examined the relationships between the variables using Spearman’s correlation and linear regression analysis. Differences between variables or datasets were assessed using Spearman’s *ρ* test. Means were compared using a one-way ANOVA and Student’s *t*-test.

## 3 Results

**Figure 2:**
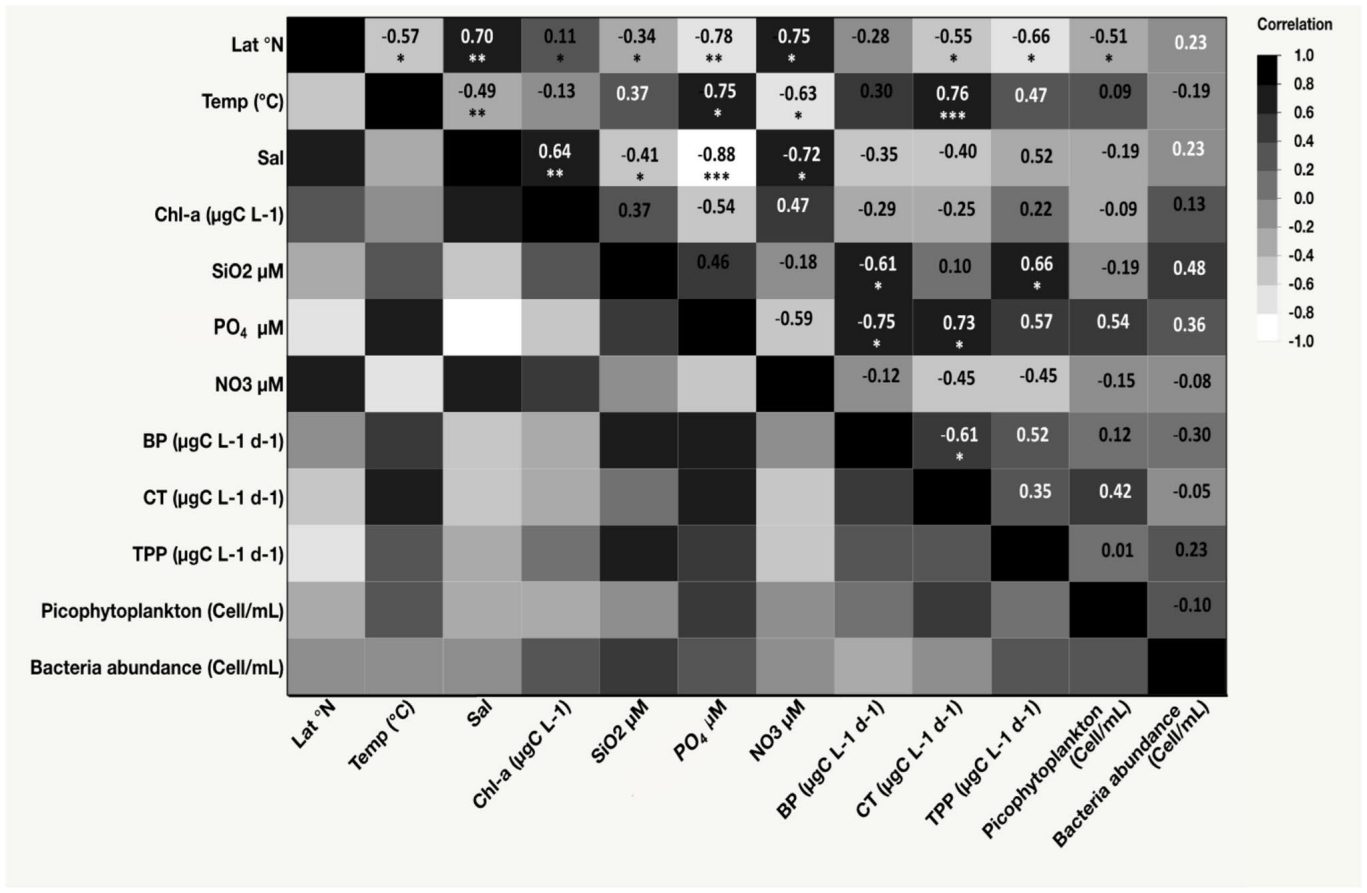
Heat map displaying Spearman’s *ρ* correlation coefficients among latitude (Lat), salinity, temperature (Temp), nutrient concentrations (phosphate [PO_4_], nitrate [NO_3_], and silicate [SiO_2_]), total primary production (TPP), carbon transfer (CT), bacterial production (BP), picophytoplankton abundance, bacteria abundance, and chlorophyll-*a* concentration (Chl-*a*). *p-value < 0.05, **p-value < 0.001, ***p-value < 0.0001.

The sea-surface temperature (SST) ranged from a minimum of 23.11°C in the northern region during winter to a maximum of 32.20°C in the coastal lagoon during the fall (Table 1). The SST increased toward the southern stations, as implied by Spearman’s *ρ* test (*ρ* = −0.57, *p-value* = 0.01, Figure 2). Salinity ranged from 38.30 to 40.80 (Min-Max, Table 1) and significantly increased with increasing latitude (*ρ* = 0.70, *p*-value = 0.001, Figure 2). Nutrient concentrations were generally low, with silicate (SiO_2_) ranging from 0.59 μM to 1.03 μM, nitrate (NO_3_) from 0.13 μM to 1.04 μM, and phosphate (PO_4_) from 0.04 μM to 0.20 μM (Table 1). SiO_2_ did not exhibit any latitudinal variability, while NO_3_ significantly increased toward the northern stations during winter (*ρ* = 0.75, *p*-value = 0.009). Conversely, PO_4_ significantly decreased with increasing latitude (*ρ* = −0.78, *p*-value = 0.004). PO_4_ tended to increase with rising temperature (*ρ* = 0.75, *p*-value = 0.008), while NO_3_ exhibited an inverse relationship with temperature (*ρ* = −0.63, *p*-value = 0.03, Figure 2).

**Table 1:** Geographical position of the sampled stations and the environmental and biological parameters obtained from various cruises (DC, DCS, RSDE, and coastal stations—lagoon, reef, and pelagic).

Cruise	Lat °N	Measurements	SST ^0^C	SiO_2_ μM	NO_3_ μM	PO_4_ μM	Chl-*a* μg l^−1^	*Synech* Cells mL^−1^	BACT Cells mL^−1^
DC	18.67	BP, CT, TPP	26.90	1.03	0.13	0.20	0.19	7.74E + 03	7.54E + 05
	19.21	BP, CT, TPP	27.00	0.95	0.21	0.19	0.18	2.04E + 04	7.84E + 05
	20.23	BP, CT, TPP	26.30	0.87	0.45	0.14	0.07	3.09E + 04	3.54E + 05
	20.75	BP, CT, TPP	26.10	0.88	0.60	0.16	0.04	1.38E + 04	6.56E + 05
	24.46	BP, CT, TPP	24.00	0.89	0.20	0.06	0.04	1.15E + 04	5.14E + 05
DCS	23.78	BP, CT, TPP, BR, BGE	24.27	0.68	0.27	0.03	0.60	4.47E + 04	5.27E + 05
	25.29	BP, CT, TPP, BR, BGE	23.72	0.89	0.83	0.05	0.48	2.17E + 04	5.23E + 05
	25.40	BP, CT, TPP, BR, BGE	24.30	0.59	0.84	0.01	0.37	6.38E + 04	5.40E + 05
	25.50	BP, CT, TPP, BR, BGE	23.11	0.83	1.04	0.04	0.62	2.34E + 04	5.54E + 05
	25.75	BP, CT, TPP, BR, BGE	23.86	0.87	0.82	0.07	0.47	1.08E + 05	6.57E + 05
	22.30	BP, CT, TPP, BR, BGE	24.90	1.03	0.59	0.11	0.49	4.33E + 04	4.38E + 05
RSDE	22.41	BP, CT, BP _Without grazers_	27.94	N/A	N/A	N/A	0.09	1.60E + 04	1.08E + 05
	22.74	BP, CT, BP _Without grazers_	27.78	N/A	N/A	N/A	0.05	1.40E + 03	5.87E + 04
Coastal	22.31 (pelagic)	BP, CT	30.40	N/A	N/A	N/A	0.50	N/A	N/A
	22.32 (Reef)	BP, CT	29.90	N/A	N/A	N/A	0.40	N/A	N/A
	22.39 (Lagoon)	BP, CT	32.20	N/A	N/A	N/A	0.70	7.29E + 04	5.44E + 05

The table presents data on the sea-surface temperature (SST), nutrient concentrations (SiO_2_, NO_3_, and PO_4_), chlorophyll-*a* concentration (Chl-*a*), *Synechococcus* (*Synech*), and bacterial abundance (BACT). Measurements from each station are listed as follows: bacterial production (BP), carbon transfer (CT), bacterial production measured without grazers (BP _Without grazers_), total primary production (TPP), bacterial respiration (BR), and bacterial growth efficiency (BGE). TPP ranged from a minimum of 2.69 μg C L^−1^ d^−1^ at the center of the Red Sea to a maximum of 16.71 μg C L^−1^ d^−1^ at the southern station, with an average of 6.21 ± 1.05 μg C L^−1^ d^−1^ throughout the study (Supplementary Table S1). TPP tended to be lower in the northern stations, exhibiting a significant negative correlation with latitude (*ρ* = −0.66, *p*-value = 0.01, Figure 2). While TPP showed no correlation with Chl-a concentration or temperature, it exhibited a significant positive correlation with SiO_2_ (*ρ* = 0.66, *p*-value = 0.02) and no correlation with NO_3_ or PO_4_.

The average concentration of phytoplankton chlorophyll-*a* was 0.31 ± 0.05 μg L^−1^ (mean ± SE), exhibiting no latitudinal variability. The highest value (0.70 μg L^−1^) was recorded in the coastal lagoon in the central Red Sea (Table 1). Picophytoplankton cell abundance (1.61 × 10^4^ ± 4.67 × 10^3^ cells mL^−1^) was dominated by the cyanobacteria *Synechococcus* sp. (Table 1). *Prochlorococcus* sp. was not detected in the surface water studied. Additionally, picophytoplankton abundance exhibited no correlation with latitude, Chl-*a* concentration, or temperature. Heterotrophic bacterial cell abundance averaged 4.89 × 10^5^ ± 6.04 × 10^4^ cells mL^−1^ and showed no latitudinal pattern or significant relationship with temperature or Chl-*a* concentration.

### 3.1 Bacterial production and carbon transfer

**Figure 3:**
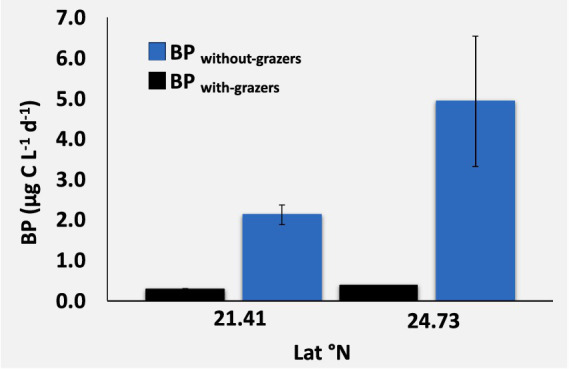
Mean and standard error for bacterial production with grazers (BP _with grazers_, black bars) and without grazers (BP _without grazers_, blue bars), comparing data from the same stations.

The BP rate for the size fraction below 1.2/3 μm, collected on 0.2 μm filters and measured as a ^13^C-glucose uptake rate, showed a 200-fold range from a minimum of 0.02 μg C L^−1^ d^−1^ to a maximum of 4.93 μg C L^−1^ d^−1^, which was recorded in the Northern Red Sea after removing grazers (Supplementary Table S1). We observed a significant correlation between BP and PO_4_ (*ρ* = 0.75, *p*-value = 0.007, Figure 2). Data comparison from the same stations revealed that removing grazers through prefiltration before incubation resulted in a much higher BP (3.52 ± 1.04 μg C L^−1^ d^−1^) compared to BP in samples that included grazers (0.37 ± 0.04 μg C L^−1^ d^−1^; one-way ANOVA, *F* = 9.08, degree of freedom (df) = 1, *p*-value = 0.02, Figure 3). These results highlighted that TPP was significantly higher than BP throughout the study (F ratio = 28.48, df = 1, *p-value* < 0.0001).

**Figure 4:**
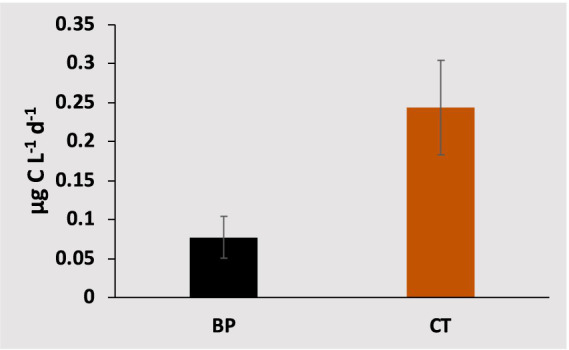
Average values (± standard error) for bacterial carbon production (BP, black bar) and carbon transferred to the food web (CT, orange bar), measured from the same incubations.

**Figure 5:**
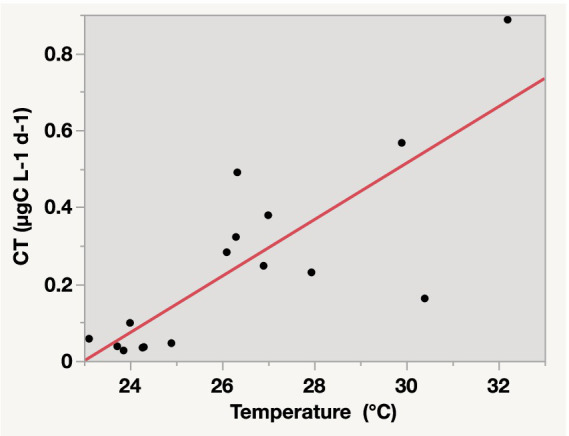
Linear relationship between carbon transfer (CT) to higher trophic levels in the microbial food web and temperature in surface waters of the Red Sea. The equation for the relationship is CT = −1.687876 + 0.0733824 × temperature, with R^2^ = 0.65 and *p-value* < 0.0001.

The CT to higher trophic levels from the incubated communities ranged from 0.03 μg C L^−1^ d^−1^ in the Central and Northern Red Sea to a maximum of 0.89 μg C L^−1^ d^−1^ in the lagoon coastal station. The CT exhibited significant average values (*F* = 6.31, df = 1, *p*-value = 0.01) compared to the BP measured from the same incubations (Figure 4; Supplementary Table S1). Additionally, CT contributed an average of 72.73% ± 4.0% to the total net BP, calculated as BP + CT. The CT declined with increasing latitude (*ρ* = −0.55, *p*-value = 0.02, Figure 2) but increased with higher PO_4_ concentrations (*ρ* = 0.73, *p*-value = 0.01, Figure 2), BP (*ρ* = 0.61, *p*-value = 0.01, Figure 2), and temperature (R^2^ = 0.65, *p*-value < 0.0001, Figure 5).

### 3.2 Bacterial respiration and bacterial growth efficiency

We measured BR during winter at six stations in the Northern Red Sea (Figure 1), which showed an average of 1.14 ± 0.54 μg C L^−1^ d^−1^ (Supplementary Table S1). BR consistently exceeded the total net BP from those stations, calculated as BP + CT (net BP, 0.06 ± 0.004 μg C L^−1^ d^−1^). Gross BP, calculated as BP + CT + BR, ranged from 0.45 to 3.76 μg C L^−1^ d^−1^, indicating that most assimilated carbon from those stations was respired rather than incorporated into biomass. BGE, calculated as (BP + CT) / (BP + CT + BR), averaged 9.7% ± 1.0% (range: 1–18%) and tended to increase with higher net BP and PO_4_ concentrations (R^2^ = 0.59, *p* < 0.01, BGE = −0.116893 + 3.3232976 × net BP; R^2^ = 0.71, *p*-value = 0.01, BGE = 0.0218532 + 1.4569595 × PO_4_).

## 4 Discussion

Our results, obtained through the addition of glucose labeled with the stable isotope ^13^C and measured using a CRDS Picarro instrument, confirmed the hypothesis that BP in the oligotrophic Red Sea efficiently contributes to CT to higher trophic levels through the microbial loop. We employed the methodology described by López-Sandoval et al. (2019), using ^13^C-NaHCO_3_ and CRDS to measure TPP in oligotrophic waters. Additionally, we successfully implemented the ^13^C-glucose method described by Koshikawa et al. (1996), following the isotopic measurement protocol by Alothman et al. (2023) using CRDS. Most studies measuring BP were based on the incorporation of radioactive isotopic labeling substrates, such as ^3^H-thymidine (Fuhrman and Azam, 1982; Kirchman et al., 1982) into nucleic acids and ^3^H-Leucine (Kirchman et al., 1985; Simon and Azam, 1989) into proteins. However, BP measurements based on labeled glucose uptake (Koshikawa et al., 1996; Koshikawa et al., 1999; Popendorf et al., 2011; Lammers et al., 2017) are less commonly used, but they provide valuable insights into direct carbon incorporation by bacteria. Glucose, an organic carbon substrate commonly produced from phytoplankton photosynthetic final products and rapidly consumed by marine bacteria, can account for up to 40% of bacterial carbon production (Skoog et al., 1999; Kirchman et al., 2001). Therefore, glucose uptake rates have been used to evaluate bacterial carbon production in marine environments (Alonso and Pernthaler, 2006; Alonso-Sáez and Gasol, 2007). Several studies have demonstrated that SAR11, the most abundant heterotrophic bacterial clade in ocean surface waters, does assimilate glucose in natural environments. For instance, Malmstrom et al. (2005) reported that SAR11 contributes 45–57% of total glucose uptake in marine waters. Additionally, Malmstrom et al. (2005) and Alonso and Pernthaler (2006) showed that both SAR11 and Roseobacter dominate glucose uptake at low nanomolar concentrations. SAR11 is a phylogenetically highly diversified lineage and certain SAR11 subgroups exhibit limited glucose assimilation, although in those cases other major bacterial taxa, such as Roseobacter and Cytophaga-Flavobacteria, present in the water actively incorporated glucose as an energy source (Alonso and Pernthaler, 2006; Teeling et al., 2012).

Given its widespread uptake by key bacterial groups, glucose uptake rates have been extensively used to evaluate bacterial carbon production and microbial carbon transfer in marine systems (Alonso and Pernthaler, 2006; Alonso-Sáez and Gasol, 2007). These findings support the ecological relevance of glucose as a valid tracer for quantifying bacterial production and carbon transfer within the microbial food web, particularly in oligotrophic marine environments like the Red Sea.

We acknowledge that glucose uptake methods have limitations, as noted by Alonso and Pernthaler (2006) and other studies. However, we selected this method based on its robust application to quantify metabolic rates particularly in oligotrophic systems where labile DOC, such as glucose plays a major role in bacterial carbon cycling (e.g., Koshikawa et al., 1996; Koshikawa et al., 1999; Middelburg et al., 2000). In addition, the ^13^C trace method used in this study required initial glucose concentrations of 75 and 100 nM, which exceeds those used in some metabolic studies (Alonso and Pernthaler, 2006) but falls below the concentrations used in other studies (Koshikawa et al., 1996; Koshikawa et al., 1999). Notably, the concentration of dissolved combined neutral monosaccharides in the Northern Red Sea, with glucose accounting for 43–52%, was approximately 0.57–0.66 μM, yielding expected glucose concentrations ranging from ~200 to 300 nM (Grossart and Simon, 2002). Therefore, the ^13^C-glucose concentrations added to the experiments in this study were within the anticipated range for the Red Sea, ensuring ecological relevance. Furthermore, we included careful experimental controls and short incubation times (4–6 h) to minimized potential biases and to avoid artifacts related to organic matter degradation from higher trophic levels. While glucose uptake may include both energy use and biosynthesis, our aim was to capture total carbon incorporation, which aligns with our study’s focus on overall bacterial production, transfer and respiration.

In our study, some sampling in the Northern Red Sea was conducted during winter, when strong winds and water column mixing increased the NO_3_ concentrations with an observed increase in the Chl-*a* concentration. These water characteristics disrupted the typical latitudinal patterns described in the Red Sea (Weikert, 1987). However, temperature gradients continued to rise toward the south. Given that Chl-*a* and temperature were uncorrelated, we analyzed the responses to temperature and trophic degree changes separately in this study. The TPP in our study ranged 2.69–16.71 μg C L^−1^ d^−1^ and overlapped with previous measurements in the same region (0.81–3.64 μg C L^−1^ d^−1^, 7.13 ± 1.35 μg C L^−1^ d^−1^, and 0.58 μg C L^−1^ d^−1^, 0.02 to 3 μg C L^−1^ h^−1^) for the Red Sea (López-Sandoval et al., 2019; Weisse and MacIsaac, 2000; Qurban et al., 2014), although our values were generally higher. While no relationship was found between TPP and picophytoplankton abundance, a relationship was observed with silicate levels. This absence of a relationship suggests that other phytoplankton species, such as diatoms, may have contributed to the TPP than picophytoplankton (Kheireddine et al., 2017).

BP values were approximately ten times lower than TPP, consistent with other studies, such as those reported in the compilation of data by Cole et al. (1988) and regarding the Red Sea (Grossart and Simon, 2002). Additionally, our BP rates (0.02–4.93 μg C L^−1^ d^−1^) fell within the range reported for the central Red Sea, the Gulf of Aden, Northern Red Sea, and Gulf of Aqaba, which were 0.58–1.80 μg C L^−1^ d^−1^, 0.29–1.08 μg C L^−1^ d^−1^ (Weisse, 1989), 0.02–1.36 μg C L^−1^ d^−1^, and 1.53 μg C L^−1^ d^−1^ (Grossart and Simon, 2002; Mescioglu et al., 2019), respectively. The D-glucose-^13^C_6_ tracer was introduced as a controlled organic carbon tracer for carbon heterotrophic uptake. Any DOC released from lysed phytoplankton would likely consist of unlabeled organic carbon rather than ^13^C-enriched, resulting in undetectable changes in the δ^13^C of samples. Additionally, in oligotrophic environments, passive phytoplankton lysis was found to be generally low during short incubations unless stress factors are present (Thornton, 2014).

We found a positive correlation between BP and PO_4_ concentration, which points that PO_4_ availability may control BP in the Red Sea, consistent with observations in other oligotrophic seas, such as the Mediterranean waters, where BP increased when phosphorus was added (Sebastián et al., 2012). Additionally, silicate had a significant positive correlation with both BP and TPP, indicating that silicate availability may increase TPP (López-Sandoval et al., 2021) as an essential nutrient for some phytoplankton species, such as diatoms, thus increasing the dissolved organic matter required for BP.

We observed a higher BP_without grazers_, measuring approximately ten times higher rate (3.52 ± 1.04 μg C L^−1^ d^−1^) than the BP_with grazers_ (0.37 ± 0.04 μg C L^−1^ d^−1^), indicating a possible high grazing pressure on bacteria by the selective fraction size at the studied Red Sea stations. Our findings provide evidence of a strong link between the microbial loop and the energy transferred to the classical food web, which was the high CT measured from the community above the 1.2/3 μm fraction size compared to BP. The higher rate of CT relative to BP reflected a high level of predation or grazing pressure on bacteria, facilitating more rapid CT to higher trophic levels and bypassing bacterial production.

Our results showed a substantial amount of carbon in the selected fraction, mainly attributed to the heterotrophic nanoflagellates (HNF) and their grazers, significantly exceeding daily BP and contributing an average of 72.73% ± 4.0% to the total net BP. BP is a pivotal component within aquatic ecosystems and serves as a primary prey source for upper trophic levels, such as flagellates. As flagellates and other grazers feed on bacteria, they facilitate the transfer of nutrients and energy to higher trophic levels, establishing a vital link between the microbial loop and the conventional food chain (Azam et al., 1983).

Consequently, recognizing the profound impact of these ecological processes on the dynamics of DOC production and nutrient recycling within aquatic ecosystems is essential. Weisse (1989) identified HNF as the primary grazers of bacteria in the oligotrophic Red Sea, with a grazing rate of 0.11 h^−1^, corresponding to a maximum of 5.4 × 10^4^ bacteria cells mL^−1^ h^−1^. This rate was notably higher in the central region than in the Gulf of Aden of the Red Sea (Weisse, 1989). Another study conducted in the Southern Red Sea examined the dynamics of the CT among various microbial loop components (bacteria, nanoflagellates, and ciliates) and found that nanoflagellates ingested 8–87 bacteria cells h^−1^ (El-Serehy et al., 2020). Additionally, ciliates were found to consume both bacteria and nanoflagellates, with ingestion rates ranging from 6 to 44 × 10^3^ bacteria cells h^−1^ and 0.2–1.85 × 10^2^ flagellate cells h^−1^ (El-Serehy et al., 2020). These findings, along with our results, support the significance of the microbial loop in the waters of this region. Thus, in such an oligotrophic ecosystem, CT through bacteria to higher trophic levels—either by grazing on bacteria or nutrient recycling—is expected to be significant (Weisse, 1989; El-Serehy et al., 2020).

Our results present evidence that grazing on bacteria is relatively high in oligotrophic regions as expected (Sherr et al., 1986). This finding aligns with previous studies reporting high grazing rates of bacteria in the Red Sea (Weisse, 1989; El-Serehy et al., 2020). Weisse (1989) reported ingestion rates of bacteria (21–58 cells HNF^−1^ h^−1^) for the central Red Sea and Gulf of Aden, which were high and within the range observed in other oligotrophic regions (10–80 bacteria HNF^−1^ h^−1^; Sherr et al., 1986). Recently, El-Serehy et al. (2020) reported even higher ingestion rates of 8–87 bacteria HNF^−1^ h^−1^ in the Southern Red Sea. Based on the measured bacterial carbon content and abundance data obtained in our study, we estimated that each bacterial cell had a carbon content of about 76.8 fg C cell^−1^. This carbon content is significantly higher than that reported in other oceanic and coastal marine systems, which averaged 12.4 ± 6.3 fg C cell^−1^, with a maximum of 30.2 ± 12.3 fg C cell^−1^ (Fukuda et al., 1998). This discrepancy may be due to the fact that we did not remove detritus and small phytoplankton, as was done in their study. Considering the calculated bacterial carbon content from our study and an HNF population of about 1,000 HNF ml^−1^ reported for the Red Sea (Weisse, 1989), which ingests 21–58 bacteria HNF^−1^ h^−1^, we calculated a CT rate ranging 0.03–0.14 μg C L^−1^ d^−1^, which is within range but lower than the maximum rate measured in our study (0.03–0.89 μg C L^−1^ d^−1^). However, if we consider an average of 10,000 HNF mL^−1^ reported in the Southern Red Sea by El-Serehy et al. (2020) and a grazing range of 8–87 bacteria HNF^−1^ h^−1^, we obtained a CT range of 0.10 to 1.60 μg C L^−1^ d^−1^, which is even higher than the range we obtained in our study.

During mesocosm experiments in a eutrophic coastal area of Japan, Koshikawa et al. (1996) quantified the percentage CT based on ^13^C-glucose uptake rate by bacteria and found that the bacteria-based food web contributed to the carbon flow into metazooplankton to an extent similar to the phytoplankton-based food web (^13^C-bicarbonate). Additionally, short incubations with ^13^C-glucose labeled highlighted that bacteria contributed approximately 6–41% to photosynthesis rates at higher trophic levels, with the ratio increasing with bacterial dominance rising (Koshikawa et al., 1999). Although the phytoplankton-based food web efficiency calculated as CT to mesozooplankton was 22%, the bacterial-based food web efficiency was only 2% in a study conducted in the northern Baltic Sea (Berglund et al., 2007). However, these results did not include the trophic levels of flagellates and ciliates, which are likely to play a significant role in carbon flow from bacteria (Berglund et al., 2007) and are expected to exceed contributions to mesozooplankton. Therefore, we may anticipate more significant CT from bacteria in oligotrophic and warm waters in the fraction size of the community above the bacteria fraction, including the trophic levels of flagellates and ciliates.

However, there is ongoing debate regarding whether the CT observed in the upper segment of the community is solely due to grazing on bacteria. Some species of diatoms, classified as autotrophic algae, can utilize various organic compounds under low light or dark conditions as part of a mixotrophic or heterotrophic life strategy, thus leveraging organic matter when photosynthesis is limited (Tuchman et al., 2006; Selosse et al., 2017; Marella et al., 2021). Research conducted by Znachor and Nedoma (2010) illustrated that organic carbon utilization by phytoplankton is of limited significance under natural conditions, as glucose is predominantly consumed by bacteria and only becomes a significant resource for diatoms when provided at high concentrations (micromolar levels). The glucose concentration in our samples was possibly insufficient to effectively support diatom uptake. However, bacteria attached to phytoplankton is another critical component within the larger community fraction that can contribute to the utilization of organic carbon (Znachor and Nedoma, 2010). However, free-living (FL) bacteria below 3 μm represent a much larger component than particle attached. A study conducted in upwelling and oligotrophic waters in North-West Africa found that free-living (FL) bacteria, below 3 μm, were two to three orders of magnitude more abundant than particulate-attached bacteria (PA) measured above 3 μm (Bachmann et al., 2018). Bachmann et al. showed that a higher abundance of PA bacteria was observed only in nutrient-rich and coastal environments. Additionally, short incubation times (4–6 h) reduce the likelihood of significant particle attachment or release during the experiment. According to these data, we assume that the contribution of PA is limited in the open-water stations sampled in our study. To the best of our knowledge, no data exist regarding the potential contribution of diatoms to dark glucose uptake or FL versus attached bacterial contributions to production in the larger fraction of the community in the Red Sea. According to these data, we assume that the contribution of PA is limited in the open-water stations sampled in our study. To the best of our knowledge, no data exists regarding the potential contribution of diatoms to dark glucose uptake or FL versus attached bacterial contributions to production in the larger fraction of the community in the Red Sea. Importantly, our glucose uptake measurements capture total carbon flow, and any PA contribution would still reflect the microbial loop’s overall carbon cycling.

Our results also indicated a strong relationship between temperature and CT, with temperature accounting for 65% of the changes in CT. This finding suggested an immediate effect of temperature on the transfer of bacterial carbon to the microbial food web in oligotrophic seas (Sarmento et al., 2010; Šolić et al., 2018), such as the Red Sea. The Red Sea is an ideal model for expected global warming scenarios in the 21st century, as reported by the IPCC (2013). It is currently experiencing a notable increase in temperature, with an average warming rate of 0.17°C ± 0.07°C per decade, surpassing the global warming rate (Chaidez et al., 2017). These findings highlight the rapid warming of the Red Sea, which could pose future challenges to its ecosystems and communities (Chaidez et al., 2017). A study by Sarmento et al. (2010) pointed that increasing warming could increase grazing on bacteria, thereby promoting bacterial carbon production flux within the microbial food web. Another study on the Adriatic Sea showed that protozoa grazing on picoplankton exceeded cell lysis as temperatures increased, indicating that increasing global warming associated with climate change could further enhance CT to higher trophic levels by picoplankton (Šolić et al., 2018).

Aside from BP, bacteria perform respiration as a function of DOC transformation. Measuring BGE helps elucidate the relationship between BP and BR and its impact on the carbon cycle and flux (Del Giorgio and Cole, 1998). In this study, we used the uptake of ^13^C-labeled glucose to measure BR and BP, as this method captures both anabolic (biosynthesis) and catabolic (energy-generating) processes. While the use of glucose as a model organic substrate may raise concerns about accurately representing natural DOC utilization, it provides valuable insights into bacterial metabolic dynamics by simulating labile carbon uptake, a key component of DOC pools. Moreover, previous studies have demonstrated that glucose uptake correlates with overall microbial metabolic patterns in oligotrophic systems, making it a suitable proxy for BP and BR assessments in environments where labile carbon plays a significant role (Smith and Prairie, 2004). Our data from the Northern Red Sea showed that BR exceeded BP by over one order of magnitude, indicating that bacteria consume organic matter at a rate greater than they produce. This suggests a net loss of dissolved organic matter and biomass (Del Giorgio and Cole, 1998) in the Northern Red Sea. The average BGE of 9.7% ± 1.0% was low and seemed to follow the BP activity pattern, consistent with the relationship found in oligotrophic Mediterranean coastal waters studied by González-Benítez et al. (2019). A lower BGE present evidence that a smaller proportion of the organic matter consumed by bacteria is used for bacterial growth and reproduction, while a larger proportion is lost through respiration and other metabolic processes (Del Giorgio and Cole, 1998). In other words, BP has a lower conversion efficiency of organic matter into new biomass. BGE is a key parameter in understanding microbial food web dynamics and biogeochemical cycles in aquatic and terrestrial ecosystems. A lower BGE can significantly impact ecosystem functioning, potentially reducing the transfer of energy and matter up the food chain and altering nutrient cycling and carbon sequestration rates (Smith and Prairie, 2004). Supporting this notion, the same northern stations where BR was measured showed the lowest CT averaged values (0.04 ± 0.004) among all the studied stations in the Red Sea (Supplementary Table S1). Similar to BP, in our study, BGE showed a positive relationship with PO_4_, suggesting that PO_4_ availability may regulate BP and BGE in the oligotrophic Red Sea, as reported for other oligotrophic areas reported by Smith and Prairie (2004).

## 5 Conclusion

This study confirms the hypothesis that heterotrophic BP in the oligotrophic Red Sea efficiently contributes to CT through the microbial loop to higher trophic levels. Using stable isotope ^13^C as a tracer, the CRDS Picarro instrument, and fractionated filtration, we successfully measured the TPP, BP, and CT in the higher trophic levels of the oligotrophic Red Sea. Our findings show that the CT through bacteria to the larger fractionated size of the microbial food web (CT > 1.2–3 μm) exceeded BP, contributing 73% of the net BP, and significantly increased with increasing water temperature, with temperature explaining 65% of the CT variability. Therefore, these findings highlight the growing significance of BP and its contribution to carbon flux and transfer through the microbial loop toward the warmest waters. Additionally, our findings suggest that BGE and BP in oligotrophic waters are regulated by PO₄ availability. The higher rate of BR relative to BP in the Northern Red Sea indicates that a large proportion of the carbon assimilated by bacteria is converted into respiration. This highlights the necessity of considering both BP and BR when evaluating the functioning of aquatic ecosystems. These findings underscore the importance of bacterial carbon dynamics in understanding carbon flow in oligotrophic marine ecosystems. Future research should explore how environmental stressors, such as temperature anomalies and nutrient fluctuations, influence BP, BGE, and BR across spatial and seasonal scales. Additionally, our approach can inform ecosystem models and carbon budgets, helping to predict how oligotrophic marine systems respond to environmental changes and contribute to carbon sequestration.

## Data Availability

The original contributions presented in the study are included in the article/Supplementary material, further inquiries can be directed to the corresponding author.
