# Supplementary material for: Flow of heterotrophic production in oligotrophic ocean waters
Source: Front Microbiol. 2025 Mar 12;16:1530627. doi: 10.3389/fmicb.2025.1530627 (PMC11938127; doi:10.3389/fmicb.2025.1530627)
Supplement: Supplementary file 1 [file Data_Sheet_1.DOCX]

Supplementary Material

# Supplementary Figure

**Figure S1:** **Schematic representation of the microbial carbon flow tracer experiment using stable isotope ^13^C.** Bacteria incorporate ^13^C from labeled D-glucose ^13^C_6_​, and the increase in δ^13^C is used to quantify bacterial production and respiration in the smaller fraction size (<3 µm). The larger fraction (>3 µm) represents carbon transfer to protists through microbial grazing.

# Supplementary Table

Supplementary Table 1: Mean ± SE of total primary production (TPP), bacterial production (BP), and carbon transfer (CT), in addition to bacterial respiration (BR) and bacterial growth efficiency (BGE), measured from different stations along the Red Sea. NA is standard for not available data.

| Cruise | Lat  (°N) | Lon  (°E) | TPP  µg C L^-1^ d^-1^ | BP  µg C L^-1^ d^-1^ | CT  µg C L^-1^ d^-1^ | BR  µg C L^-1^ d^-1^ | BGE  % |
| --- | --- | --- | --- | --- | --- | --- | --- |
| DC | 18.67 | 40.22 | 16.71± 0.59 | 0.04 ± 0.01 | 0.25 ± 0.01 | NA | NA |
|  | 19.21 | 39.95 | 6.08 ± 0.48 | 0.08 ± 0.04 | 0.38 ± 0.00 | NA | NA |
|  | 20.23 | 39.26 | 4.99 ± 0.24 | 0.02 ± 0.00 | 0.32 ± 0.07 | NA | NA |
|  | 20.75 | 39.09 | 6.22 ± 0.67 | 0.06 ± 0.01 | 0.28 ± 0.05 | NA | NA |
|  | 24.46 | 37.09 | 2.98 ± 0.46 | 0.02 ± 0.00 | 0.10 ± 0.01 | NA | NA |
| DCS | 22.30 | 38.86 | 14.74 ± 4.44 | 0.04 ± 0.00 | 0.05 ± 0.01 | 0.36 | 18 % |
|  | 23.78 | 37.65 | 10.37 ± 1.86 | 0.02 ± 0.00 | 0.03 ± 0.00 | 1.17 | 4 % |
|  | 25.29 | 37.65 | 4.83 ± 0.94 | 0.03 ± 0.00 | 0.04 ± 0.02 | 0.43 | 13 % |
|  | 25.40 | 36.50 | 3.17 ± 0.28 | 0.02 ± 0.00 | 0.04 ± 0.01 | 3.76 | 1 % |
|  | 25.50 | 36.40 | 3.18 ± 0.87 | 0.03 ± 0.00 | 0.06 ± 0.01 | 0.57 | 12 % |
|  | 25.75 | 36.34 | 3.28 ± 0.44 | 0.03 ± 0.01 | 0.03 ± 0.01 | 0.49 | 9 % |
| RSDE | 22.41 | 38.67 | 1.54 ± 0.05 | 2.12 ± 0.19 | NA | NA | NA |
|  | 22.41 | 38.67 | 1.54 ± 0.05 | 0.29 ± 0.03 | 0.23 ± 0.00 | NA | NA |
|  | 24.72 | 36.27 | 5.84 ± 0.01 | 0.39 ± 0.03 | 0.49 ± 0.03 | NA | NA |
|  | 24.72 | 36.27 | 3.38 ± 1.53 | 4.93 ± 0.03 | NA | NA | NA |
| Coastal | 22.31  (pelagic) | 38.96 | 3.47 ± 0.20 | 0.02 ± 0.00 | 0.16 ± 0.02 | NA | NA |
|  | 22.32  (reef) | 39.02 | 4.34 ± 0.35 | 0.12 ± 0.01 | 0.57 ± 0.06 | NA | NA |
|  | 22.39  (lagoon) | 39.14 | 15.90 ± 6.38 | 0.04 ± 0.01 | 0.89 ± 0.42 | NA | NA |
